# Single-cell RNA sequencing reveals the vascular smooth muscle cell phenotypic landscape in aortic aneurysm

**DOI:** 10.1186/s12964-023-01120-5

**Published:** 2023-05-15

**Authors:** Genmao Cao, Xuezhen Xuan, Yaling Li, Jie Hu, Ruijing Zhang, Haijiang Jin, Honglin Dong

**Affiliations:** 1 Department of Vascular Surgery, The Second Hospital of Shanxi Medical University, No. 382, Wuyi Road, Taiyuan, China; 2 Department of Nephrology, The Second Hospital of Shanxi Medical University, No. 382, Wuyi Road, Taiyuan, China

**Keywords:** Vascular smooth muscle cells, Phenotypes, Single-cell transcriptome analysis, Aortic aneurysm

## Abstract

**Background and objectives:**

Phenotypic switching in vascular smooth muscle cells (VSMCs) has been linked to aortic aneurysm, but the phenotypic landscape in aortic aneurysm is poorly understood. The present study aimed to analyse the phenotypic landscape, phenotypic differentiation trajectory, and potential functions of various VSMCs phenotypes in aortic aneurysm.

**Methods:**

Single-cell sequencing data of 12 aortic aneurysm samples and 5 normal aorta samples (obtained from GSE166676 and GSE155468) were integrated by the R package Harmony. VSMCs were identified according to the expression levels of ACTA2 and MYH11. VSMCs clustering was determined by the R package ‘Seurat’. Cell annotation was determined by the R package ‘singleR’ and background knowledge of VSMCs phenotypic switching. The secretion of collagen, proteinases, and chemokines by each VSMCs phenotype was assessed. Cell‒cell junctions and cell–matrix junctions were also scored by examining the expression of adhesion genes. Trajectory analysis was performed by the R package ‘Monocle2’. qPCR was used to quantify VSMCs markers. RNA fluorescence in situ hybridization (RNA FISH) was performed to determine the spatial localization of vital VSMCs phenotypes in aortic aneurysms.

**Results:**

A total of 7150 VSMCs were categorize into 6 phenotypes: contractile VSMCs, fibroblast-like VSMCs, T-cell-like VSMCs, adipocyte-like VSMCs, macrophage-like VSMCs, and mesenchymal-like VSMCs. The proportions of T-cell-like VSMCs, adipocyte-like VSMCs, macrophage-like VSMCs, and mesenchymal-like VSMCs were significantly increased in aortic aneurysm. Fibroblast-like VSMCs secreted abundant amounts of collagens. T-cell-like VSMCs and macrophage-like VSMCs were characterized by high chemokine levels and proinflammatory effects. Adipocyte-like VSMCs and mesenchymal-like VSMCs were associated with high proteinase levels. RNA FISH validated the presence of T-cell-like VSMCs and macrophage-like VSMCs in the tunica media and the presence of mesenchymal-like VSMCs in the tunica media and tunica adventitia.

**Conclusion:**

A variety of VSMCs phenotypes are involved in the formation of aortic aneurysm. T-cell-like VSMCs, macrophage-like VSMCs, and mesenchymal-like VSMCs play pivotal roles in this process.

Video Abstract

**Supplementary Information:**

The online version contains supplementary material available at 10.1186/s12964-023-01120-5.

## Introduction

Aortic aneurysm (AA) is defined as permanent local aortic expansion that exceeds the normal diameter by more than 50% [1]. Previous studies have shown that the direct mechanical cause is a decrease in active contractile force and a reduction in elastic recoil force caused by damage to the structural integrity of the aorta and apoptosis in VSMCs. Pathological analysis revealed that macrophages and lymphocytes infiltrate the aortic wall, resulting in chronic inflammatory conditions. During this process, proinflammatory cells secrete excessive amounts of matrix metalloproteinases (especially MMP2 and MMP9) that decompose ECM proteins such as collagen and elastin into fragments, resulting in ECM remodelling. Intraluminal thrombus (ILT) can be detected in the majority of AA cases, and high levels of cytokines, proteases, and ROS in ILT accelerate AAA progression [2, 3].

There are currently no guidelines or recommendations for effective pharmacological treatments for AA, and surgical repair is required when the AA diameter exceeds 5.5 cm [4]. This is because many drug trials targeting the traditional pathogenesis of AA have failed. Doxycycline, a nonselective MMP inhibitor, was not effective in inhibiting AAA growth in a randomized controlled trial involving 282 patients [5]. Similarly, trials investigating fenofibrate (targeting atherosclerosis) and ticagrelor (targeting intraluminal thrombus) failed to reduce aortic aneurysm expansion [6, 7].

In recent years, the development of single-cell transcriptome and lineage-tracing techniques has revealed the pivotal role of VSMCs phenotypic switching in vascular disease. For instance, in chronic kidney disease (CKD) patients, calcium-phosphate metabolism disorder triggers contractile VSMCs to switch into osteoblast-like VSMCs, contributing to vascular calcification [8]. Macrophage-like VSMCs can engulf oxLDL and debris that contribute to the lipid-rich necrotic cores of atherosclerotic plaques [9]. Synthetic/secretory VSMCs are involved in neointima formation after vascular injury [10]. However, the phenotypes of VSMCs in AA remain unclear. The expression of contractile marker proteins (αSMA, SM22α, CNN1) is decreased in AA tissue, indicating the phenotypic transformation of contractile VSMCs [11]. Therefore, the present study aimed to identify the potential phenotypic landscape of VSMCs in aortic aneurysm by reanalysing single-cell sequencing data. The characteristics and functions of the identified phenotypes were determined, and the developmental trajectories of VSMCs phenotypes were analysed. This study provides novel insight into the pathogenesis of aortic aneurysm.

## Methods

### General analyses of single-cell sequencing data

Single-cell transcriptome data of the aortic aneurysm datasets GSE155468 (including 8 aortic aneurysm samples and 3 normal aorta samples) and GSE166676 (including 4 aortic aneurysm samples and 2 normal aorta samples) were integrated by the ‘Harmony’ package [12]. The datasets were analysed using ‘Seurat’ v4.1.1 in R [13, 14]. Genes that were expressed in fewer than 10 cells were excluded from subsequent analysis. The following cells were excluded: 1) percent of mitochondrial genes was greater than 5%; 2) number of feature genes was less than 200; and 3) number of read counts was less than 2000. Raw counts from each cell were normalized by the total counts in that cell, multiplied by a million, and then natural-log transformed by adding a pseudocount of 1. The top 2000 highly variable genes (HVGs) were selected regarding mean expression and variance. Seurat objects were scaled by the percentage of mitochondrial genes and sample identity. Then, the top 50 principal components were calculated among 2000 HVGs for PCA dimensionality reduction. A two-dimensional uniform manifold approximation and projection (UMAP) plot was used to visualize the first 20 principal components. A shared nearest neighbour (SNN) graph was constructed using 20 dimensions of the principal components. Differential gene expression between clusters was examined using the Wilcox test implemented in Seurat, and each gene was required to be expressed in at least 50% of cells in either of the two groups.

### VSMCs subset analysis and annotation

All cells with ACTA2 expression levels > 1 and MYH11 expression levels > 1 were selected from the total cell population. The selected cells were then used to create a new Seurat object and compute the HVGs. The expression matrix was scaled and normalized, and then an SNN graph was established by 20 principal components. Finally, we adjusted the resolution parameter so that the number of VSMCs clusters obtained was 10–15.

This study used the computational tool ‘singleR’ for cell cluster annotation, which is based on comparing annotated reference data with chosen marker genes that optimally discriminate cell phenotypes [15]. Manual annotations relying on rich background knowledge of biology were also performed. At present, there is no unified definition of the potential phenotypes of VSMCs. Since we intended to identify the presence of synthetic VSMCs, macrophage-like VSMCs, osteoblast-like VSMCs, mesenchymal-like VSMCs, fibroblast-like VSMCs, adipocyte-like VSMCs, and endothelial-like VSMCs in aortic aneurysm, the selected marker genes were as follows: Fibroblast-like VSMCs (collagen +) [16], macrophage-like VSMCs (CD68 + , CD14 +) [17, 18], osteoblast-like VSMCs (BMP2 + , SOX9 +) [8, 19, 20], mesenchymal-like VSMCs (SCA1/LY6A + , CD34 +) [21], adipocyte-like VSMCs (FABP4 + , PPARG +) [21, 22], and endothelial-like VSMCs (VWF + , PECAM1/CD31 +) [22, 23]. Clusters with the same phenotype were combined into one cluster. Trajectory analysis was conducted by Monocle v2.24.1 [24].

### Functional analysis

Previous studies have shown that VSMCs undergo phenotypic transformation in vascular diseases and secrete a large number of extracellular matrix proteins such as collagen [25], matrix metalloproteinases such as MMP-2 [17], and inflammatory chemokines [17]. Therefore, we evaluated the expression levels of related genes in each VSMCs phenotype with a heatmap. Gene lists of collagen, proteinases, and chemokines were obtained from Li et al. [26]. To further analyse the changes in VSMCs subtypes, GO functional enrichment analyses were conducted on upregulated genes (log (fold change) > 0.25) in each annotated VSMCs phenotype by ClusterProfiler v3.14.3 (https://guangchuangyu.github.io/software/clusterProfiler/). The cell‒cell junction score and cell–matrix junction score were calculated to assess the internal structure compactness of the aorta. The calculation formulas can be found in Li et al. [26].

### SCENIC analysis

SCENIC analysis was performed by referring to the motif databases RcisTarget and GRNboost. The R packages SCENIC v1.3.1 [27], AUCell v1.18.1, and RcisTarget v1.16.1 were used.

Then, the RcisTarget package was used to analyse overrepresented transcription factor (TF) binding motifs. After the Spearman’s correlation between the TF and the potential targets was calculated, the coexpressed genes for each TF were established by the ‘runGenie3’ function. The runSCENIC function generated the GRNs (termed regulons). Finally, the AUCell package was used to calculate the regulon activity scores of each group of regulons in each cell.

### Mouse model of abdominal aortic aneurysm

C57/BL6 mice (male, 8–10 weeks old) were purchased from the Experimental Animal Center of Shanxi Medical University. After anaesthetization by ether inhalation, a ventral midline incision was made through the abdominal skin and muscles. The colon and intestine were exposed and gently moved to the right abdomen region and covered with 0.9% saline-soaked gauze. The abdominal aorta was carefully isolated from the inferior vena cava under an optical microscope. Subsequently, a gelatine sponge (1 mm * 1 mm * 5 mm) dipped in elastase solution (100 mg/ml) was closely apposed on the infrarenal aorta for 20 min. The control group was treated with a gelatine sponge dipped in 0.9% saline. After removing the sponge, 0.9% saline at 37 °C was used for peritoneal lavage three times. Muscle and skin were sutured with 6–0 nylon sutures following reset of the abdominal organs. The mice were placed in a thermostatic chamber for postoperative recovery. The mice were fed 0.2% (v/v) 3-aminopropionitrile fumarate (BAPN). Twenty-one days later, laparotomy was performed to observe the formation of AA. The aortic aneurysm model was considered to be successful if the expansion rate of the aortic diameter exceeded 50%.

### *RNA fluorescence *in situ* hybridization and quantitative polymerase chain reaction*

For RNA fluorescence in situ hybridization (RNA FISH), normal aortic or aortic aneurysm tissue was fixed in 4% paraformaldehyde for at least 12 h and dehydrated in a sucrose solution. The tissue sections were prehybridized in hybridization buffer at 37 °C for 1 h and then hybridized with probes (probe sequences are in Table S1 in the Data Supplement). Images were observed with a scanning confocal microscope (Pannoramic DESK, 3DHistech, Hungary).

For quantitative polymerase chain reaction (qPCR), a standard procedure was performed. Total RNA was extracted using the Tiangen RNA Simple Total RNA Kit (DP419, Tiangen). Subsequently, 1 µg of total RNA was reverse transcribed using PrimeScript RT Master Mix (RR036A, Takara). Amplification was performed using SYBR Green Premix (RR420A, Takara). NADPH was used as the internal reference. The primer sequences were as follows: ACTA2: F-GTCCCAGACATCAGGGAGTAA, R-TCGGATACTTCAGCGTCAGGA; CD68: F-TGTCTGATCTTGCTAGGACCG, R-GAGAGTAACGGCCTTTTTGTGA; CD3D: F-AGCGGGATTCTGGCTAGTCT, R-CGCTGGTATTGCAGGTCACAA; and CD34: F-AAGGCTGGGTGAAGACCCTTA, R-TGAATGGCCGTTTCTGGAAGT.

### Cell‒cell communication analysis

To investigate intercellular communication strength between various VSMCs phenotypes and identify the expression of vital signalling and ligand receptors, the R package “CellChat” (version 1.1.3) was applied to all identified VSMCs phenotypes [28]. We also compared the differences in communication strength, signalling, and ligand receptors between aortic aneurysm-derived VSMCs and normal aorta-derived VSMCs.

## Results

### All cell types in aortic aneurysm

**Fig. 1:**
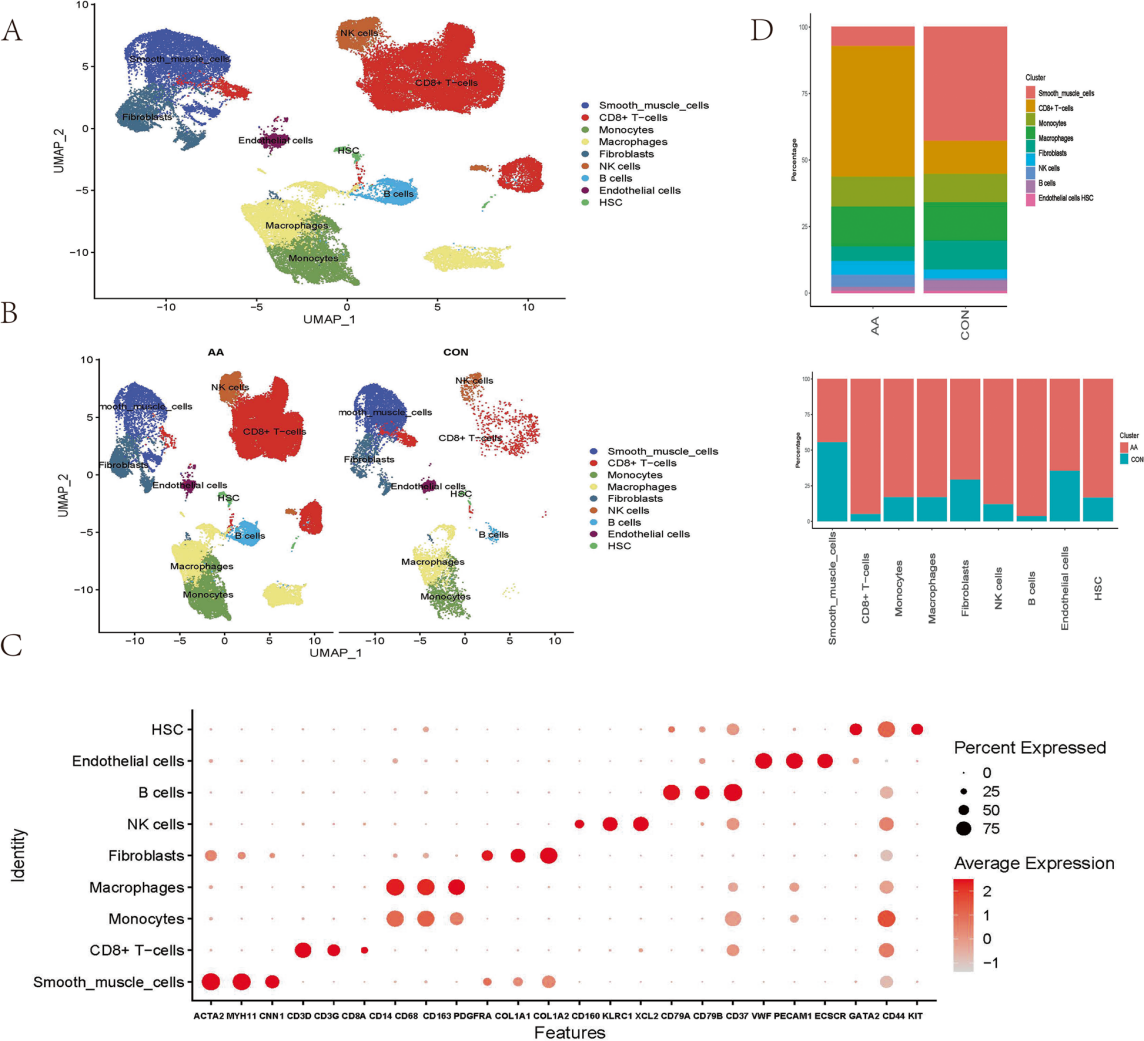
General analysis and annotation of all cells from the normal aorta and aortic aneurysm. Uniform manifold approximation and projection (UMAP) representation of the aligned gene expression data from the normal aorta and aortic aneurysm, showing the partitioning of 9 cell types **A** and groupings by sample source **B**. The expression of marker genes is shown in the dot plot **C**. The origin distribution of various cell types and the proportions of various cell types in aortic aneurysm and the normal aorta **D**

A total of 50,690 cells with 37,335 gene expression profiles were used to construct the Seurat object. An unsupervised clustering algorithm in Seurat was used for filtered cells, resulting in 25 distinct clusters. Regarding canonical markers and automated reference-based annotation tools (SingleR), the clusters were monocytes/macrophages (CD14 + CD68 + CD163 +), smooth muscle cells (ACTA2 + MYH11 + CNN1 +), endothelial cells (ECs) (PECAM1 + VWF + ECSCR +), CD8 + T cells (CD3D + CD3E + CD8A +), B cells (CD37 + CD79A + CD79B +), natural killer cells (CD160 + KLRC1 + XCL2 +), fibroblasts (COL1A1 + COL1A2 + PDGFRA +), and haematopoietic stem cells (HSCs) (KIT + CD44 + GATA2 +) (Fig. 1A-C). The proportion of immune cells (macrophages, B cells, T cells, NK cells) was increased in aortic aneurysm, while the proportions of VSMCs and fibroblasts were decreased (Fig. 1D).

### Identifying VSMCs phenotypes

**Fig. 2:**
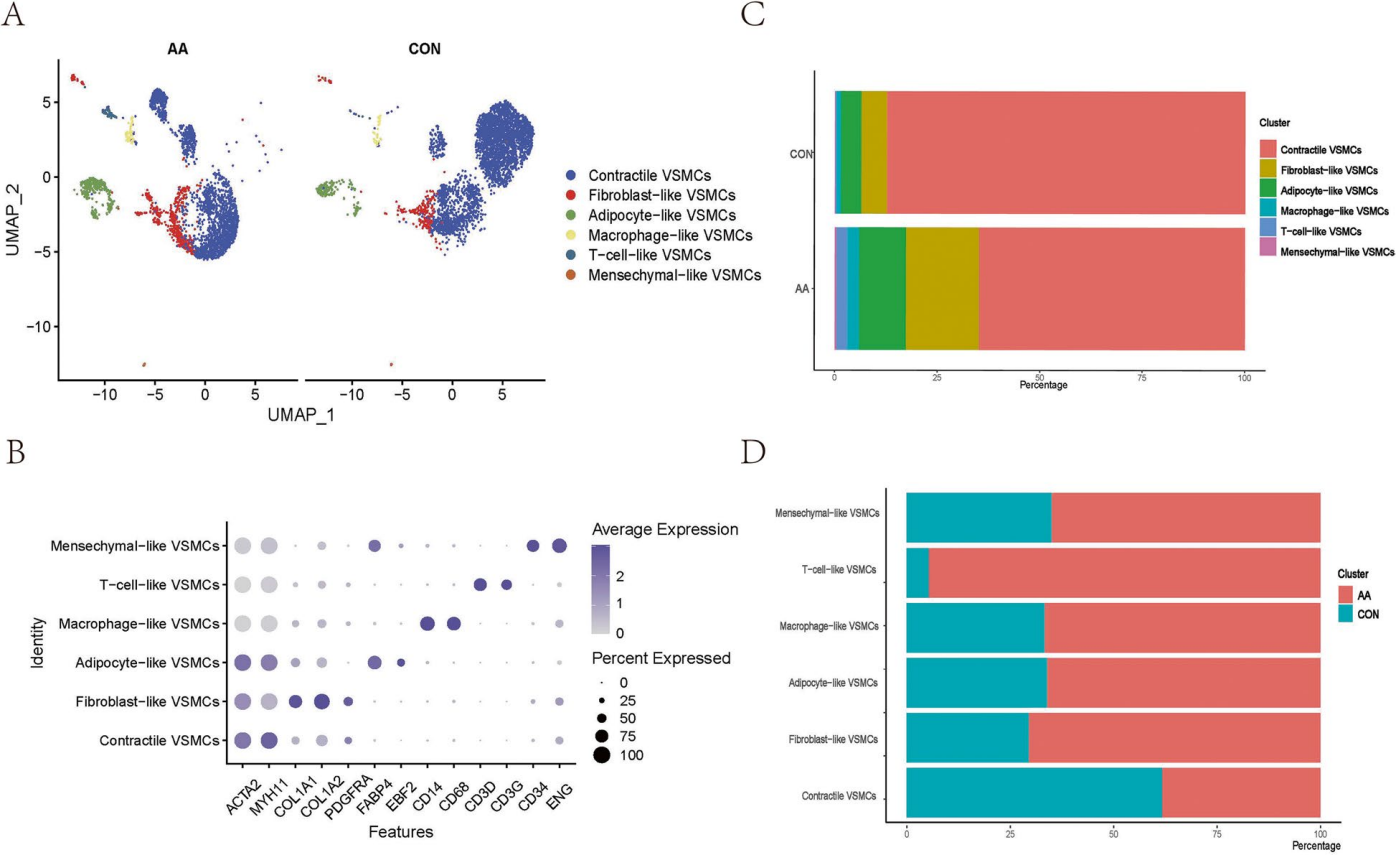
Specific analysis and annotation of all VSMCs phenotypes. Uniform manifold approximation and projection (UMAP) representation of the aligned gene expression data of all VSMCs showing the partitioning of 6 annotated VSMCs phenotypes **A**. The expression of marker genes is shown on the dot plot **B** (gene expression log-normalized by Seurat). The proportion of VSMCs phenotypes in aortic aneurysm and the normal aorta **C** and the origin distribution of various VSMCs phenotypes **D**

A total of 7150 cells were preliminarily identified as VSMCs (3255 from aortic aneurysm, 3895 from the normal aorta). These cells were clustered into 14 Seurat clusters after being scaled and normalized. By labelling canonical markers and calculating differential genes, potential VSMC phenotypes were identified as follows: (i) Contractile VSMCs highly expressed the VSMCs marker genes ACTA2 and MYH11, possessed the function of contraction and expressed no other specific markers (CD68-CD34-FABP4- CD3D-). (ii) Fibroblast VSMCs, which showed lower expression of the contraction genes ACTA2 and MYH11, robustly secreted extracellular matrix (COL1A1, COLIA2) and showed no expression of other specific markers (CD68-CD34-FABP4-CD3D-). (iii) T-cell-like VSMCs mildly expressed VSMC markers (ACTA2 + MYH11 +) and strongly expressed T-cell markers (CD3D + CD3G +), and they also exhibited the highest levels of inflammatory gene expression. (iv) Adipocyte-like VSMCs expressed adipocyte markers (EBF2 + FABP4 +) and no other specific markers (CD68-VWF-CD34-KLRB1-). (v) Macrophage-like VSMCs had high expression of macrophage markers (CD14 + CD68 +). (vi) Mesenchymal-like VSMCs highly expressed stem cell markers (CD34 + ENG +) (Fig. 2A-B).

### Functional analysis

#### Contractile VSMCs

**Fig. 3:**
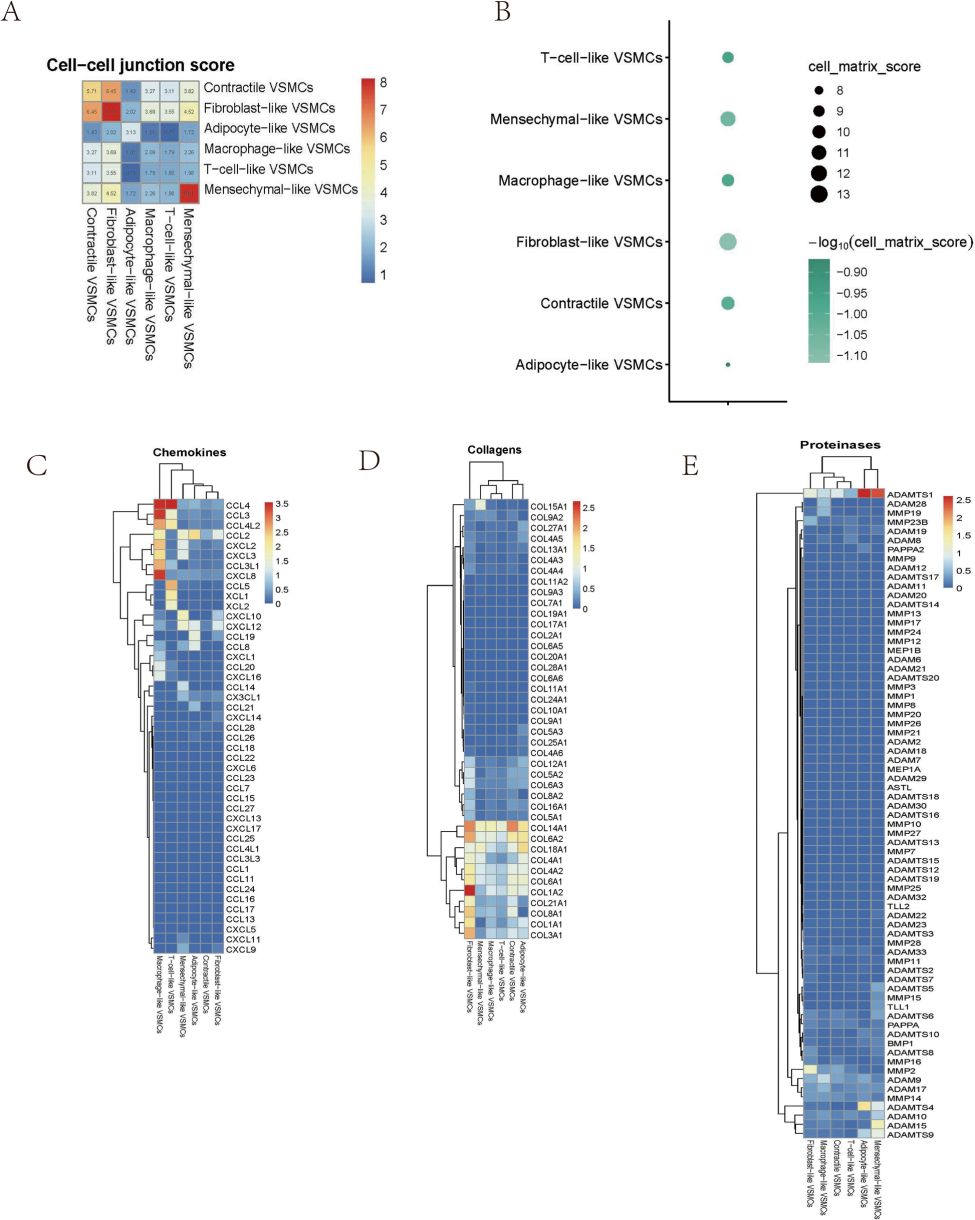
Functional analysis of various VSMCs phenotypes. The cell‒cell junction scores between any two VSMCs phenotypes **A** and cell–matrix scores of each VSMCs phenotype **B**. Heatmap showing chemokine genes **C**, collagen genes **D**, and proteinase genes **E** in various VSMCs phenotypes

Contractile VSMCs were present in aortic aneurysm and normal aortic tissue, but their proportion in AA was significantly decreased (Fig. 2C-D). This result suggested that VSMCs were less capable of contracting during aortic aneurysm and dedifferentiating into other potential phenotypes. Contractile VSMCs showed high expression of contractile proteins and exhibited high cell‒cell junction scores and cell–matrix junction scores, which are important for maintaining the structural integrity and rigidity of the aortic wall (Fig. 3A-B). The expression of inflammatory chemokines was lowest in contractile VSMCs, which was consistent with normal aorta physiology. Some ECM genes (such as COL14A1) were upregulated in contractile VSMCs to maintain the ECM structure and repair the ECM. Moreover, lower protease gene expression in contractile VSMCs was associated with less ECM remodelling in normal aortas (Fig. 3C-D). Contractile VSMCs primarily offered contractile force and constituted the muscle structure (Fig. S1A).

#### Fibroblast-like VSMCs

The proportion of fibroblast-like VSMCs was higher in aortic aneurysm than in the normal aorta (Fig. 2E-F). Fibroblast-like VSMCs also had the highest cell‒cell junction score and cell–matrix junction score (Fig. 3A-B). Compared with those in contractile VSMCs, the contractile genes in fibroblast-like VSMCs were decreased, but overall ECM gene expression was further increased (Fig. 3D). Thus, fibroblast-like VSMCs possess a stronger ability to secrete ECM proteins, which means that fibroblast-like VSMCs may mediate repair after vascular injury. The overall level of proteases in fibroblast-like VSMCs was low, but the expression of MMP2 was increased, indicating that fibroblast-like VSMCs are involved in ECM remodelling to a certain extent, which is consistent with previous studies (Fig. 3E). Interestingly, the expression levels of inflammatory chemokines (CXCL10, CXCL12, CCL2) in fibroblast-like VSMCs were slightly higher than those in contractile VSMCs (Fig. 3C). The upregulated genes in fibroblast-like VSMCs were enriched in extracellular matrix organization and collagen-containing extracellular matrix (Fig. S1B).

#### Mesenchymal-like VSMCs

An increased proportion of mesenchymal-like VSMCs was present in aortic aneurysms (Fig. 2C-D). We observed downregulated expression of contraction genes in mesenchymal-like VSMCs, and no other specific markers were expressed except for stem cell markers (CD34 + ENG +). Mesenchymal-like VSMCs obtained a moderate cell–matrix junction score and a low cell‒cell junction score (Fig. 3A-B). In addition, mesenchymal-like VSMCs exhibited moderate chemokine levels, moderate ECM secretion, and relatively high proteinase levels (Fig. 3C-E). The enriched GO terms of mesenchymal-like VSMCs were similar to those of secretory VSMCs and included extracellular matrix organization, collagen-containing extracellular matrix, and collagen binding (Fig. S1C).

#### Adipocyte-like VSMCs

Approximately 70% of adipocyte-like VSMCs were present in aortic aneurysms (Fig. 2C-D). Adipocyte-like VSMCs obtained low cell‒cell junction scores and the lowest cell–matrix junction scores (Fig. 3A-B). Adipocyte-like VSMCs were characterized by low chemokine levels but expressed CCL2, CCL8, CCL19, and CXCL12 (Fig. 3C). In addition, adipocyte-like VSMCs exhibited moderate ECM protein levels (Fig. 3D). The general expression of proteases was low except for ADAMTS1 and ADAMTS4 (Fig. 3E). Regarding gene enrichment analysis, adipocyte-like VSMCs were involved in ribosomal metabolism and peptide hormone metabolism (Fig. S1D).

#### Macrophage-like VSMCs

The proportion of macrophage-like VSMCs was significantly increased in aortic aneurysms (Fig. 2C-D). Macrophage-like VSMCs did not form strong cell‒cell junctions with any VSMC phenotype, and a moderate cell–matrix junction score was obtained (Fig. 3A-B). The most significant characteristic of macrophage-like VSMCs was that these cells had the highest level of chemokines such as CCL3, CCL4, and CXCL8 (Fig. 3C). No significant expression of ECM or protease genes was observed (Fig. 3D-E). Functional enrichment analysis indicated that macrophage-like VSMCs exerted effects on antigen processing and presentation (Fig. S1E).

#### T-cell-like VSMCs

The characteristics of T-cell-like VSMCs are similar to those of macrophage-like VSMCs. T-cell-like VSMCs were primarily present in aortic aneurysms (Fig. 2C-D). Among all VSMC phenotypes, T-cell-like VSMCs obtained the lowest cell‒cell junction scores than other VSMCs phenotypes and a low cell–matrix junction score (Fig. 3A-B). T-cell-like VSMCs were characterized by low ECM protein levels, low protease levels, and high chemokine levels (Fig. 3C-E). T-cell-like VSMCs were involved in the positive regulation of leukocytes according to GO functional enrichment analysis (Fig. S1F).

### Single-cell trajectory analysis

**Fig. 4:**
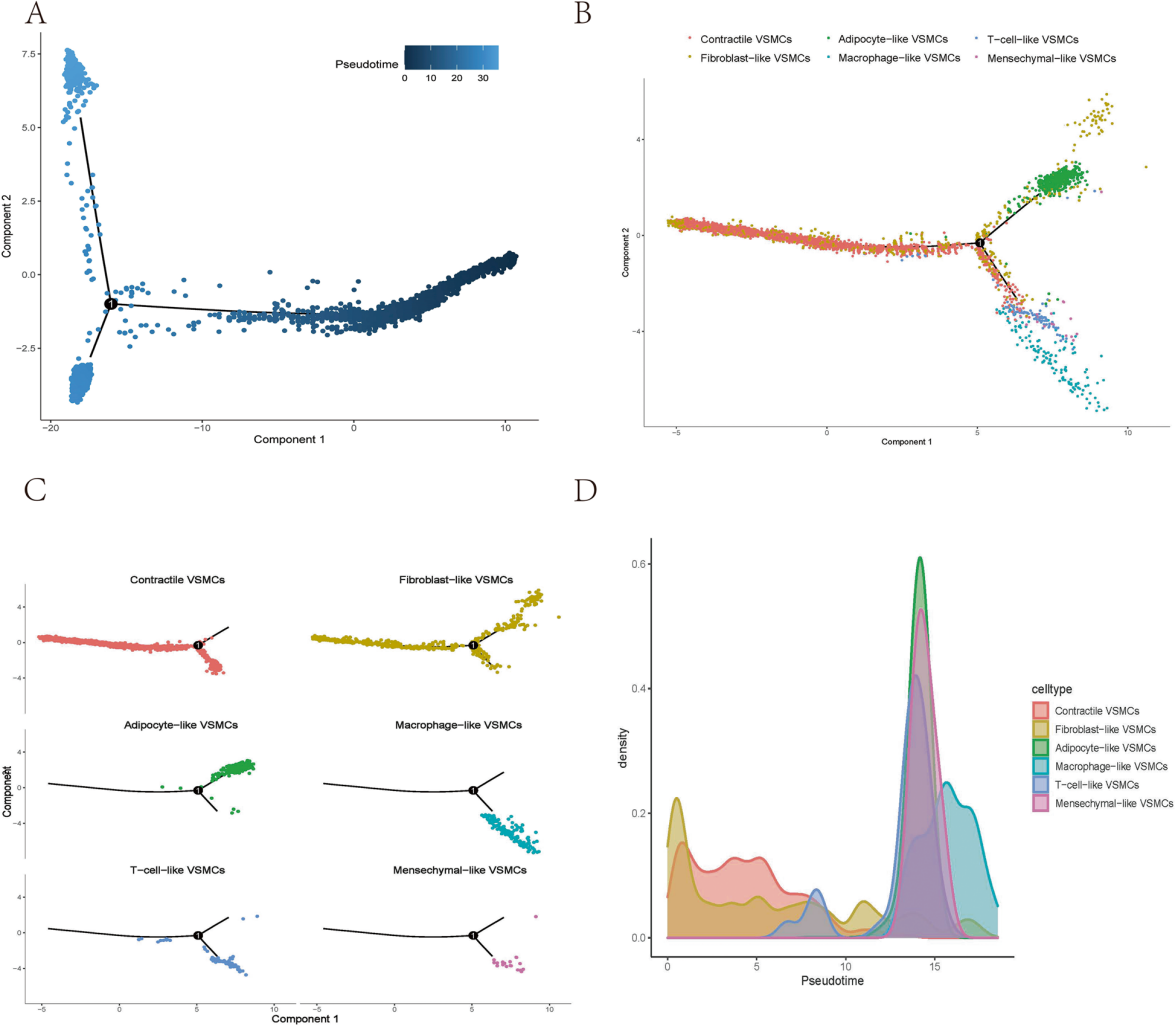
Cell trajectory analysis of various VSMCs phenotypes. Pseudotime plot showing trajectory analysis of all VSMCs **A** and various VSMCs phenotypes **B**-**C**. Cell density is plotted along the timeline **D**

To identify the origin, trajectory, and timing of differentiation in the VSMC phenotypes, we performed trajectory analysis. From the pseudotime plot and the phenotype-based trajectory plot, we learned that all VSMCs phenotypes were initially derived from contractile VSMCs and fibroblast-like VSMCs (Fig. 4A). During the mid-to-late timeline, contractile VSMCs and fibroblast-like VSMCs gradually differentiated into two groups (Fig. 4B-C). One group differentiated into T-cell-like VSMCs, macrophage-like VSMCs, and mesenchymal-like VSMCs. Fibroblast-like VSMCs appeared in the early stage of aortic aneurysm formation, which may be a response to vascular injury (Fig. 4D). T-cell-like VSMCs and macrophage-like VSMCs, which are present in the late stage, may be related to the progression of aortic aneurysm.

### Regulon network analysis by SCENIC

**Fig. 5:**
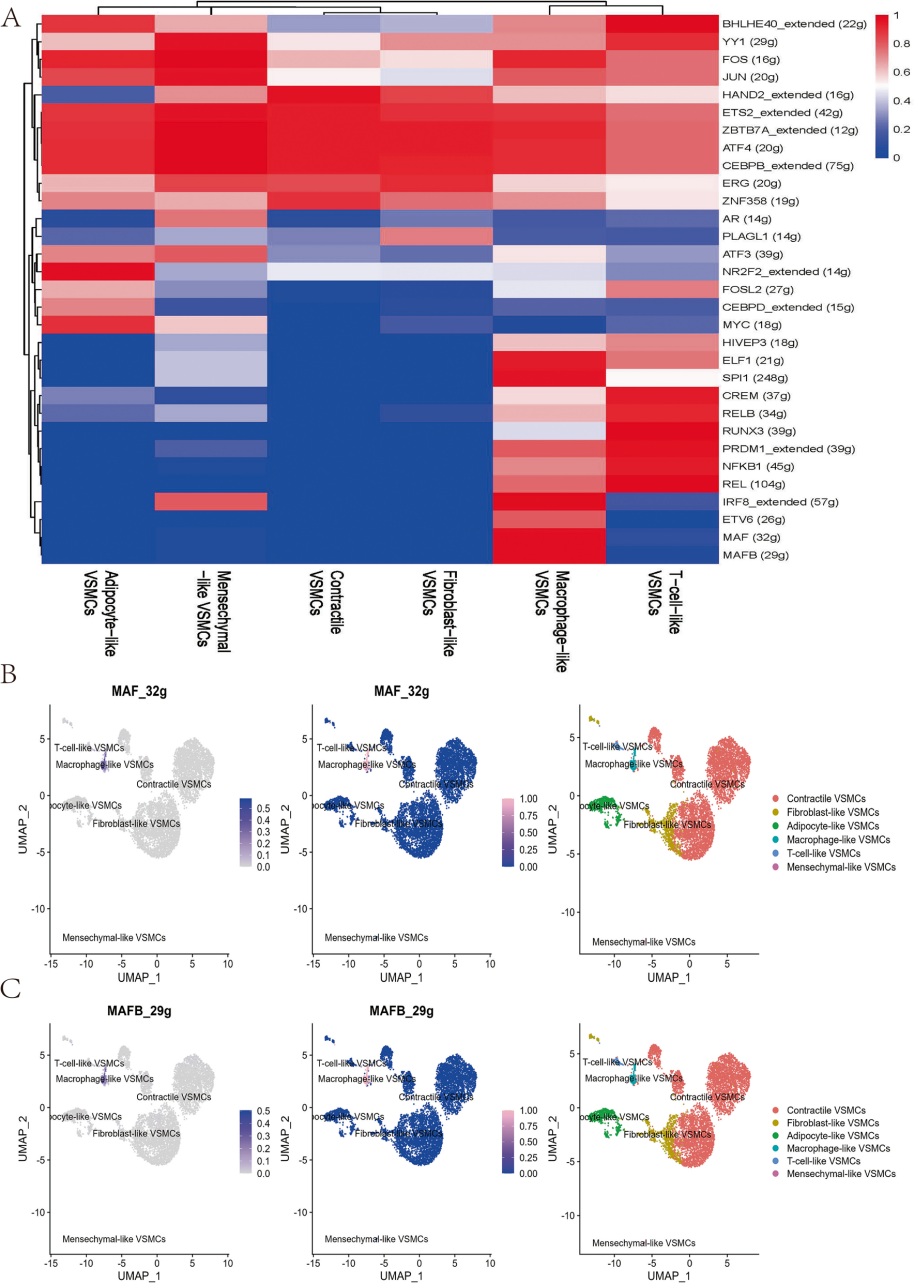
SCENIC analysis indicated significant regulons for each VSMCs phenotype **A**. The regulatory activity of MAF (32 g) **B** and MAFB (29 g) **C** was projected with a UMAP plot

Previous studies have shown that TFs such as KLF4 [21], RUNX2 [29], and SRF [30] play a pivotal role in regulating VSMC phenotypes. We further investigated the correlation between regulon (TFs and their target genes) activity and VSMC phenotypes using SCENIC. A total of 144 regulons were constructed, and 31 regulons scored high activity (Fig. 5A). MAF (32 g) and MAFB (29 g) had specific regulatory effects on macrophage-like VSMCs (Fig. 5B-C). RUNX3 (39 g) specifically regulated T-cell-like VSMC differentiation (Fig. S2A). NR2F2_extended (14 g) demonstrated high regulatory activity on adipocyte-like VSMCs (Fig. S2B). Fibroblast-like VSMCs were positively regulated by PLAGL1 (14 g), and mesenchymal-like VSMCs were positively regulated by AR (14 g) (Fig. S2C-D).

### Assessment of cell‒cell communication among 6 VSMCs phenotypes

**Fig. 6:**
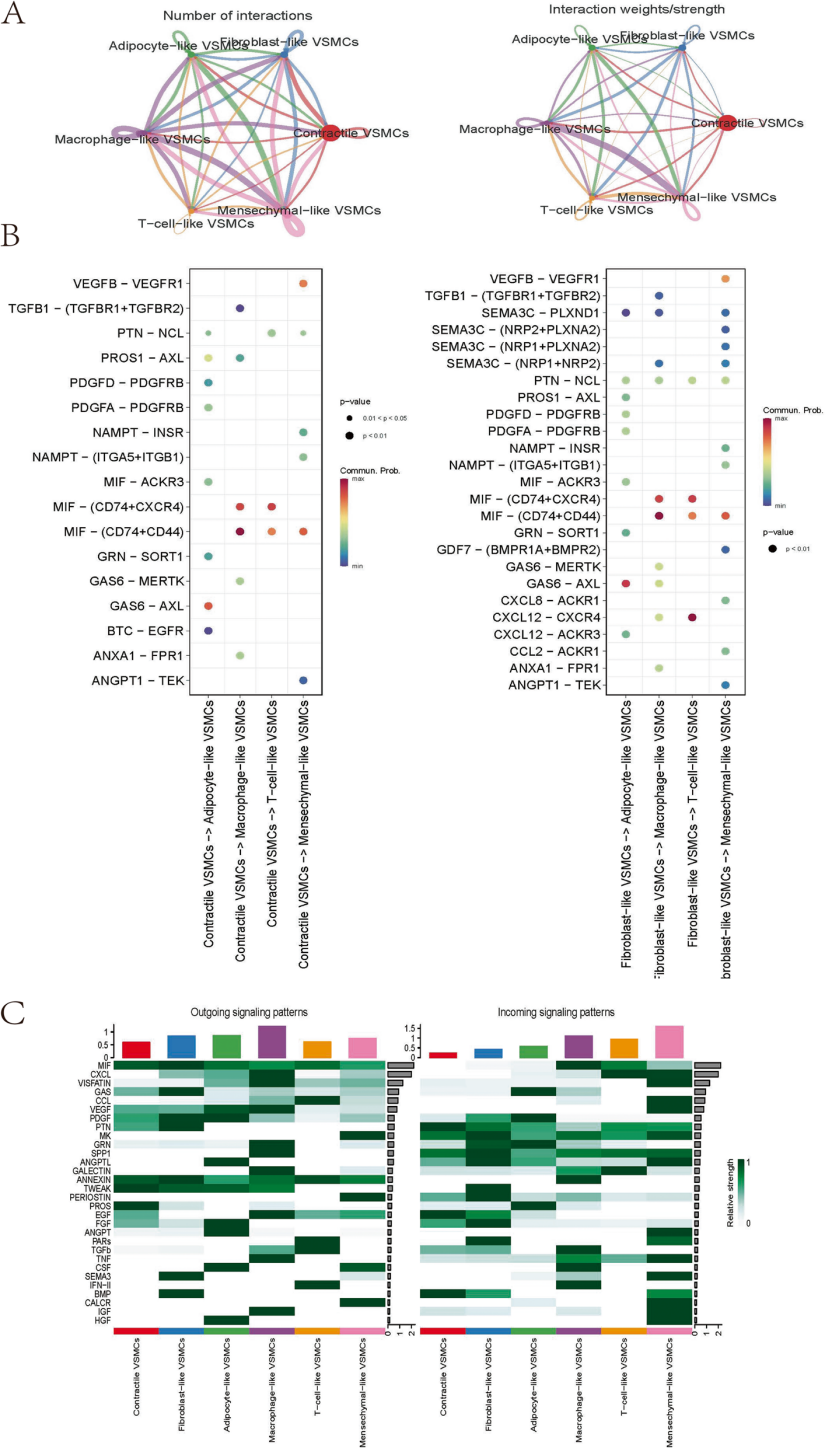
Global cell‒cell communication patterns involve multiple signalling pathways. The line width represents the interaction quantity and interaction strength among various VSMCs phenotypes **A**. Communication strength of all significant signalling pathways from contractile/fibroblast-like VSMCs to macrophage-like/T-cell-like/adipocyte-like/mesenchymal-like VSMCs **B**. Significant signalling of each VSMCs phenotype in incoming/outgoing communication patterns **C**

As previously mentioned, there is no understanding of how VSMCs phenotypes occur in aortic aneurysms. We believe that intercellular communication among distinct VSMCs phenotypes may accelerate this process. Therefore, the R package ‘CellChat’ was used to investigate the communication and interaction among identified VSMC phenotypes. We first assessed the general interactions between various VSMC phenotypes. Figure 6A shows the number of cell‒cell communications and the interaction strength among 6 VSMCs phenotypes. Macrophage-like VSMCs possessed the highest interaction numbers and strength. Pseudotime analysis showed significant signalling pathways from early-emerging VSMCs to late-occurring VSMCs (Fig. 6B). Macrophage migration inhibitory factor (MIF) signalling showed the highest level of communication probability. Figure 6C shows the incoming/outgoing communication patterns of VSMCs populations. Macrophage-like VSMCs mediated the highest incoming communication, while mesenchymal-like VSMCs mediated the highest outgoing communication.

Regarding the network central plot, contractile VSMCs, fibroblast-like VSMCs, and adipocyte-like VSMCs were more likely to act as senders in the MIF signalling-mediated network (Fig. S3A). On the other hand, macrophage-like VSMCs, T-cell-like VSMCs, and mesenchymal-like VSMCs were suggested to play sophisticated roles. In the MIF signalling network, fibroblast-like VSMCs send the most signals, which were mainly received by macrophage-like VSMCs (Fig. S3B-C). The ligand‒receptor pairs MIF-(CD74 + CXCR4), MIF-(CD74 + CD44), and MIF-ACKR3 contributed to MIF signalling (Fig. S3D).

### Quantification of VSMCs markers and the spatial localization of VSMCs

**Fig. 7:**
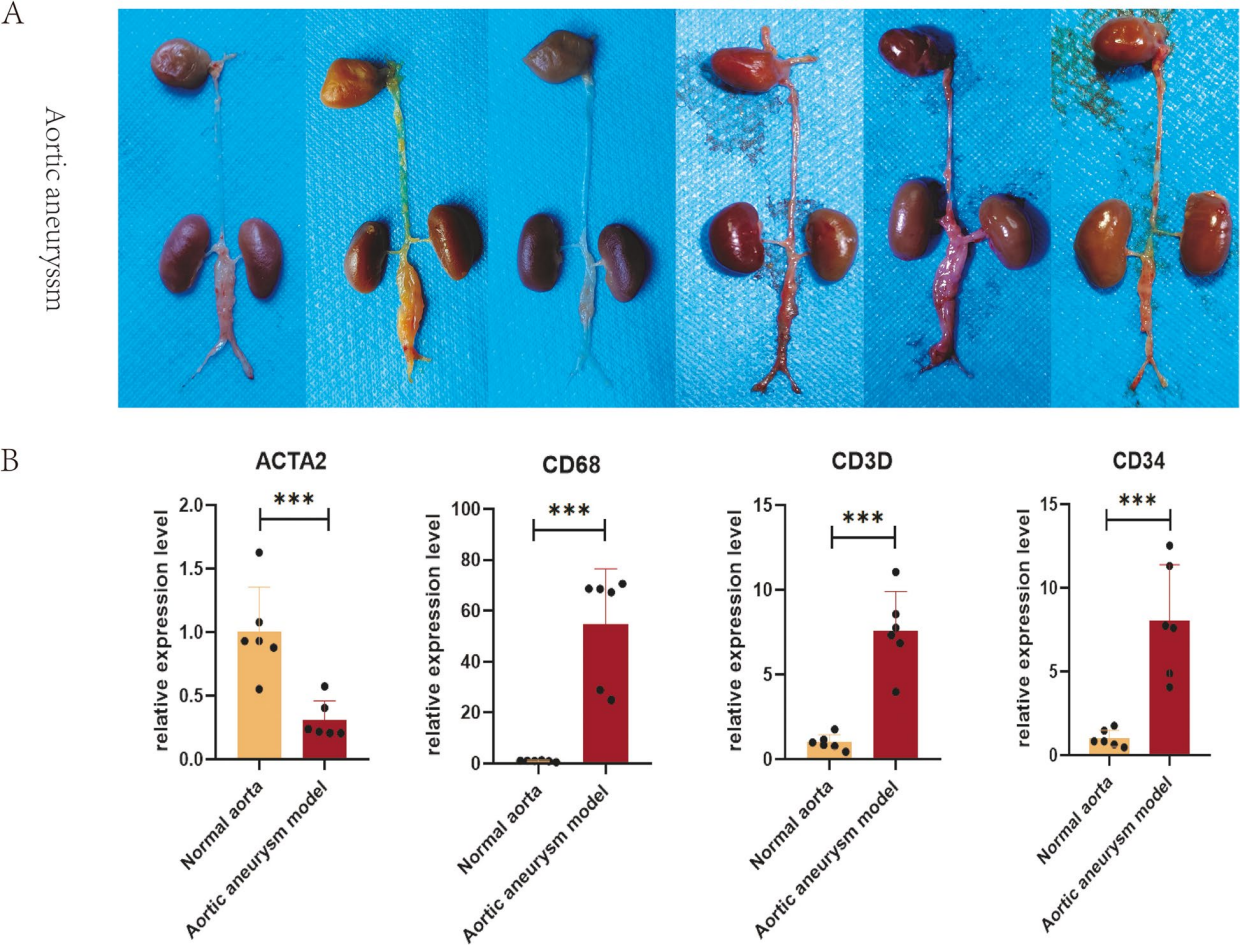
Successful construction of the mouse abdominal aortic aneurysm model **A**. The relative expression levels of ACTA2. CD68, CD3D, and CD34 were quantified by qPCR **B**

**Fig. 8:**
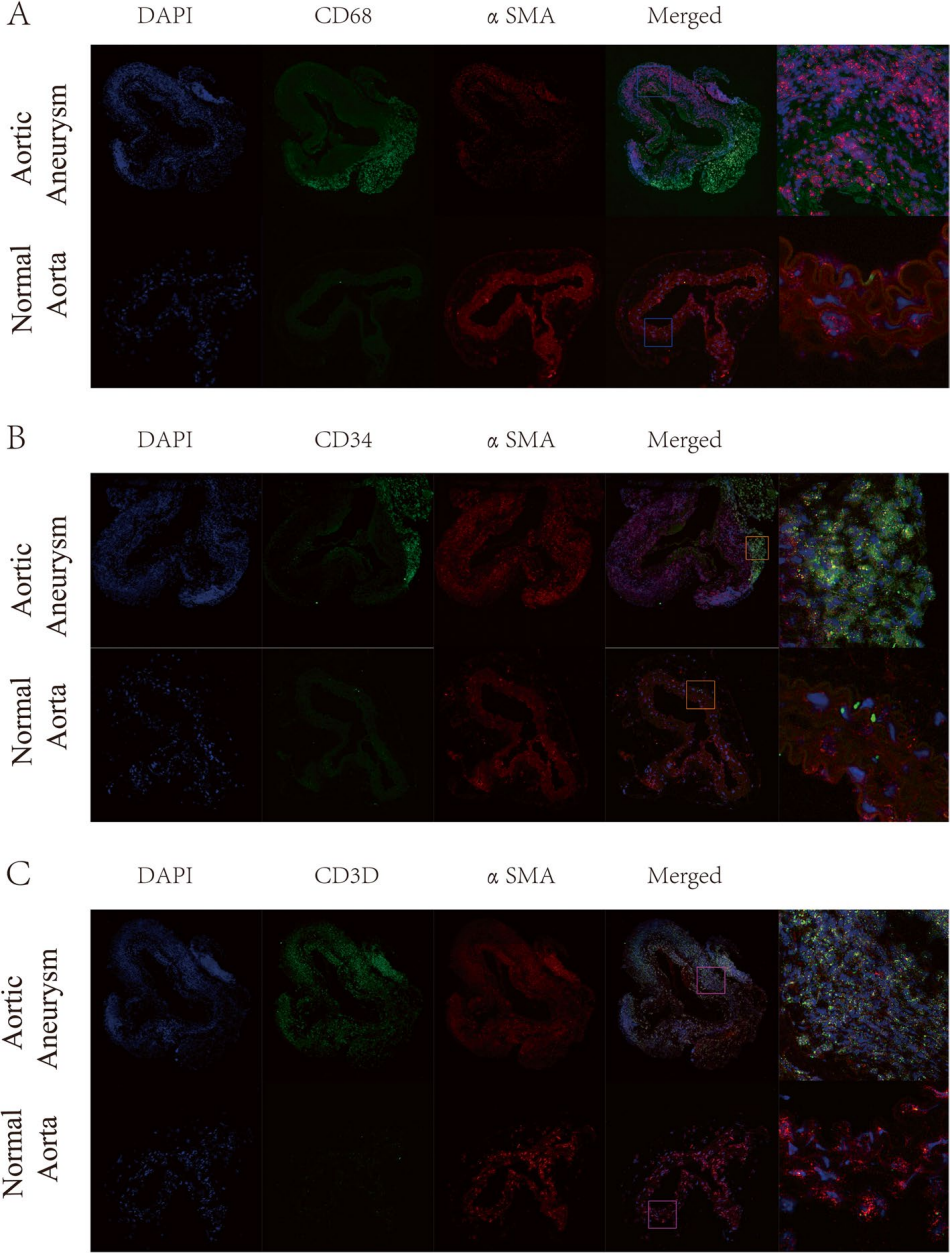
RNA FISH of the normal aorta and aortic aneurysm. Dual hybridization of αSMA and CD68 to identify macrophage-like VSMCs **A**. Dual hybridization of αSMA and CD34 to identify mesenchymal-like VSMCs **B**. Dual hybridization of αSMA and CD3D to identify T-cell-like VSMCs **C**

Mesenchymal-like VSMCs, macrophage-like VSMCs, and T-cell-like VSMCs were selected for further validation by qPCR and dual immunofluorescence analysis, due to their potential vital roles in aortic aneurysms. A mouse model of aortic aneurysm was successfully established (Fig. 7A). The relative expression level of the contractile marker ACTA2 decreased to 0.307 (*p* < 0.001) in the aortic aneurysm model, while the expression level of CD68 increased to 54.85 (*p* < 0.001) (Fig. 7B). Additionally, CD3D and CD34 were overexpressed (CD3D = 7.59 (*P* < 0.001), CD34 = 8.025 (*p* < 0.001)). RNA FISH indicated that a spot of cells coexpressing αSMA and CD68 (Fig. 8A) and a spot of cells coexpressing αSMA and CD3D were present in the tunica media of aneurysm tissue (Fig. 8C), verifying the presence of macrophage-like VSMCs and T-cell-like VSMCs. No macrophage-like VSMCs or T-cell-like VSMCs were detected in normal aortas. A few cells coexpressing αSMA and CD34 were present in normal aortas (Fig. 8B). However, the number of αSMA + /CD34 + cells and the fluorescence intensity were dramatically increased in full-thickness aortic aneurysms, indicating the transition of normal VSMCs into mesenchymal-like VSMCs.

## Discussion

In healthy adult arteries, VSMCs are in a quiescent contractile state with little proliferation or migration ability [31]. Normal VSMCs overexpress contractile proteins to form numerous myofilaments. Calcium-regulated aortic contraction occurs when the aorta dilates [32]. On the other hand, VSMCs secrete ECM proteins, which are important components for maintaining normal aorta structure. In the thoracic aorta segment, VSMCs mostly secrete elastin, while collagen is mostly secreted in the abdominal aorta segment [33]. The characteristics of contractile VSMCs in our study were consistent with those of normal VSMCs. Therefore, the reduced proportion of contractile VSMCs in aortic aneurysms indicates that normal contractile VSMCs have undergone apoptosis or phenotypic transformation. Earlier studies confirmed that VSMCs dedifferentiate into the secretory phenotype after vascular injury, which is characterized by decreased expression of contractile proteins, increased collagen synthesis, irregular morphology, an extensive rough endoplasmic reticulum, and a large Golgi complex [34]. Therefore, it is reasonable to identify Clusters 9 and 15 as secretory VSMCs. Secretory VSMCs account for a higher proportion of cells in aortic aneurysms. Although these cells synthesize more collagen to repair the structural integrity of the aorta, the contractile force of the aorta is also reduced. Thus, the aorta cannot retract to a normal diameter in response to pulse blood pressure, resulting in aortic aneurysm. A study showed that the density of focal adhesions in secretory VSMCs is reduced, resulting in reduced cell–matrix adhesion, thus making the internal structure of the aorta less compact and prone to structural remodelling [35].

In a systematic review, vascular mesenchymal cells were classified as “fibroblasts,” “myofibroblasts”, “smooth muscle cells”, “fibrocytes”, “mesangial cells”, and “pericytes” [36]. Pan et al. found a VSMCs-derived intermediate cell in atherosclerotic plaques, which was termed the ‘SEM’ cell because it could transform in a variety of directions [37]. The transformation of "SEM" cells into macrophage-like VSMCs and other VSMCs phenotypes promoted atherosclerotic plaque progression, while ATRA treatment could reverse “SEM” cells to a normal state. Because "SEM" cells express the mesenchymal markers LY6A (SCA1) and LY6C1 but do not express the mesenchymal cell markers ENG (CD105) and NT5E (CD73), Pan et al. suggested that there were differences between "SEM" cells and mesenchymal cells. In Carmen's review, SCA1 + CD34 + VSMCs were defined as mesenchymal cell-like VSMCs [21], which included the "SEM" cells in Pan et al. The two most significant terms identified by GO enrichment analysis of "SEM" cells were "Extracellular matrix organization" and "Extracellular structure organization," which is consistent with our GO analysis results of mesenchymal-like VSMCs in aortic aneurysms. Adipocyte-like VSMCs were proposed by Shankman et al. [38] because these cells were FABP4 positive. Among the identified adipose-like VSMCs, the GO enrichment pathways of upregulated genes included mesenchymal cell-related and cell junction-related pathways. Moreover, adipocytes overlapped with mesenchymal VSMCs more than other phenotypes in the cell trajectory plot. Therefore, we believe that these cells are mesenchymal VSMCs that express adipose markers.

Cell trajectory analysis indicated that macrophage-like VSMCs and T-cell-like VSMCs were the last clusters to differentiate. GO functional enrichment analysis showed that macrophage-like VSMCs activated neutrophils and that T-cell-like VSMCs activated lymphocytes. Macrophage-like VSMCs also expressed high levels of chemokines such as CCL and CXCL. We concluded that macrophage-like VSMCs and T-cell-like VSMCs could promote the progression of AA by enhancing the immune infiltration and inflammatory response of the aortic wall. The presence of macrophage-like VSMCs has been verified in atherosclerosis. In atherosclerosis, macrophage-like VSMCs are mainly located in the necrotic core and play a similar role to macrophages. In vitro, high oxLDL and cholesterol stimulate VSMCs to transition into a macrophage-like phenotype [39, 40]. Therefore, in atherosclerotic plaques, macrophage-like VSMCs phagocytose oxidized LDL and necrotic cell fragments and then convert into foam cells to participate in the formation of the lipid necrotic core. However, it was reported that macrophage-like VSMCs were less phagocytic than myeloid macrophages, and this type of macrophage slowed lipid clearance [9]. Furthermore, macrophage-like VSMCs secrete numerous inflammatory factors, such as TNF-α, IL-1B, IL-6, IL-8, IL-17, CCL2, and CCL7, leading to local inflammatory activation and exacerbating the inflammatory state [41, 42]. However, hyperlipidaemia was not one of the risk factors for aortic aneurysm, and foam cells were not involved in aortic aneurysm formation. Thus, macrophage-like VSMCs mainly participate in the formation and progression of aortic aneurysm by secreting inflammatory factors. This finding was consistent with our result that macrophage-like VSMCs express a large number of chemokines. Our results also indicated that macrophage-like VSMCs express relatively high levels of MMP2 for ECM remodelling. At present, no T-cell-like VSMCs have been reported to be present in vascular disease. T-cell-like VSMCs coexpress CD3D, CD8A, ACTA2, and MYH11. Given the absence of solid evidence provided by lineage cell tracing experiments, we cannot neglect the possibility that T-cell-like VSMCs are derived from T cells.

The cell membrane of VSMCs expresses abundant integrin-based focal adhesions that connects the nonmuscle actin cytoskeleton with ECM proteins such as collagen and elastin [43, 44]. Integrin-based focal adhesions not only mediate the transduction of biochemical signals in and out of cells but also transduce extracellular mechanical forces into the intracellular cytoskeleton, which is involved in regulating VSMC phenotypic switching [45, 46]. Sufficient focal adhesions can ensure that normal VSMCs have strong cell–matrix adhesion and cell‒cell adhesion, which means a high degree of tightness within the aorta. After VSMCs phenotypic switching, the downregulation of adhesion genes impaired cell–matrix junctions and cell‒cell junctions, leading to the loose structure of the aorta.

## Limitations

In the present study, VSMC phenotypes were identified by canonical cell markers such as ACTA2 and MYH11. However, these markers are not VSMCs specific, and endothelial-mesenchymal-transformed cells can also express ACTA2 in the disease state. Further studies of VSMCs phenotypic switching require cell lineage tracing experiments.

## Supplementary Information

**Additional file 1.** 

**Additional file 2.** 

**Additional file 3.** 

**Additional file 4.** 

## Data Availability

The datasets used and analysed in the current study are available from the public Gene Expression Omnibus database (https://www.ncbi.nlm.nih.gov/geo). Other raw data supporting the conclusions of this article will be available upon reasonable request.
